# Dependence of Working Memory on Coordinated Activity Across Brain Areas

**DOI:** 10.3389/fnsys.2021.787316

**Published:** 2022-01-13

**Authors:** Ehsan Rezayat, Kelsey Clark, Mohammad-Reza A. Dehaqani, Behrad Noudoost

**Affiliations:** ^1^School of Cognitive Sciences, Institute for Research in Fundamental Sciences (IPM), Tehran, Iran; ^2^Department of Ophthalmology and Visual Sciences, University of Utah, Salt Lake City, UT, United States; ^3^Cognitive Systems Laboratory, Control and Intelligent Processing Center of Excellence (CIPCE), School of Electrical and Computer Engineering, College of Engineering, University of Tehran, Tehran, Iran

**Keywords:** working memory, synchrony, oscillation, brain disorders, causal manipulation

## Abstract

Neural signatures of working memory (WM) have been reported in numerous brain areas, suggesting a distributed neural substrate for memory maintenance. In the current manuscript we provide an updated review of the literature focusing on intracranial neurophysiological recordings during WM in primates. Such signatures of WM include changes in firing rate or local oscillatory power within an area, along with measures of coordinated activity between areas based on synchronization between oscillations. In comparing the ability of various neural signatures in any brain area to predict behavioral performance, we observe that synchrony between areas is more frequently and robustly correlated with WM performance than any of the within-area neural signatures. We further review the evidence for alteration of inter-areal synchrony in brain disorders, consistent with an important role for such synchrony during behavior. Additionally, results of causal studies indicate that manipulating synchrony across areas is especially effective at influencing WM task performance. Each of these lines of research supports the critical role of inter-areal synchrony in WM. Finally, we propose a framework for interactions between prefrontal and sensory areas during WM, incorporating a range of experimental findings and offering an explanation for the observed link between intra-areal measures and WM performance.

## Introduction

Working memory (WM), as a basic cognitive function, contributes to our goal-oriented behaviors such as decision-making, problem-solving, language comprehension, and learning (Gazzaniga and Ivry, 2013). Persistent activity has been the traditional signature for implicating an area in WM (Kojima and Goldman-Rakic, 1982; Funahashi et al., 1989; Miller et al., 1996); however, persistent activity is rarely a strong predictor of memory performance (in terms of percent correct, accuracy, or faster reaction times; Table 1), raising questions about whether it is the best indicator of an area’s contribution to memory maintenance. Moreover, many areas show such persistent spiking activity during the delay period of a WM task (i.e., delay activity), suggesting that memory maintenance may depend on distributed activity across multiple brain areas (Christophel et al., 2017). This hypothesis leads to the question of how these active areas interact during the task.

**TABLE 1:** Neural signatures of working memory (WM) within areas and their relationship to behavior.

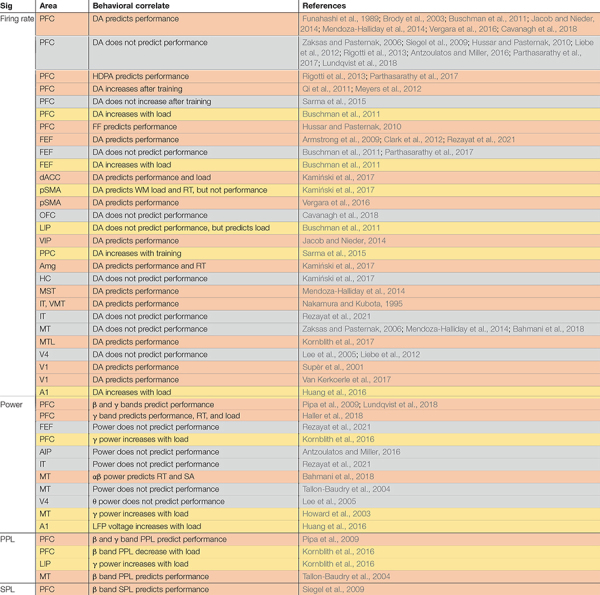

*Studies are first grouped according to the neural signature being studied during WM maintenance (Sig). Firing rate measures are usually based on single neurons, whereas LFP power, spike-phase locking (SPL), and phase-phase locking (PPL) are population-level measures. SPL measures the regularity of spike timing relative to the phase of a particular LFP oscillatory frequency. Phase-phase locking (PPL) measures synchronization between the same frequency oscillation at two sites. The second column groups studies by the area being recorded from Area. The effect of WM and its relationship between this modulation and the animal’s behavior is noted (Behavioral correlate). Each row summarizes related studies (References). Coloring indicates whether the signature was correlated with performance [percent correct, reaction time (RT), or saccade accuracy (SA); orange] or some other aspect of behavior on a WM task (load, training; yellow); rows in gray showed no correlation, blue showed negative correlation. Studies which report data for more than one area may be listed multiple times. Note that in humans, ECoG measurements of LFPs biased are toward temporal and frontal sites as a result of clinical considerations (Tallon-Baudry et al., 2001; Howard et al., 2003; Axmacher et al., 2008, 2010; van Vugt et al., 2010; Khursheed et al., 2011; Maris et al., 2011; van der Meij et al., 2012; Noy et al., 2015; Kambara et al., 2017, 2018; Myroshnychenko et al., 2017; Ni et al., 2017; Johnson et al., 2018a,b; Zhang et al., 2018; Alagapan et al., 2019b; Gehrig et al., 2019; Boran et al., 2020). Sx, signature; PFC, prefrontal cortex; lPFC, lateral PFC; dACC, dorsal anterior cingulate cortex; pSMA, pre supplementary motor area; OFC, orbitofrontal cortex; FEF, frontal eye field; LIP, lateral intraparietal; VIP, ventral intraparietal; PPC, posterior parietal cortex; HC, hippocampus; Amg, amygdala; MT, middle temporal; VMT, ventromedial temporal; MST, medial superior temporal; MLT, medial temporal lobe; IT, inferior temporal; FR, firing rate; RT, reaction time; FF, fano factor; PPL, phase phase locking; SPL, spike phase locking; DA, delay activity of single neurons; HDPA, high dimension population activity. Frequency bands (θ, α, β, and γ) are reported based on the definitions in each reference; exact cutoffs may vary, but roughly correspond to 4–8, 8–15, 15–35, and 35–80 Hz, respectively.*

Synchronized activity between brain areas provides a potential means to modulate communication during WM and other tasks (Varela et al., 2001; Fries, 2005, 2015; Sakurai and Takahashi, 2008; Canolty et al., 2010; Fell and Axmacher, 2011; Luczak et al., 2013; Canavier, 2015; Yuste, 2015; Avena-Koenigsberger et al., 2018; Singer, 2018; Hahn et al., 2019).

In this review, we summarize findings on changes in oscillatory and synchronized activity within and between brain areas during WM, including correlations with behavioral performance, impairments associated with mental disorders, and causal manipulations. Finally, we suggest a framework for interactions between prefrontal and visual areas which offers an explanation for why success in WM tasks relies on inter-areal synchrony.

## Population-Level Signatures Predict the Behavioral Performance on Working Memory Tasks

The activity of individual neurons often fails to predict WM performance. A summary of studies which reported the correlation (or lack thereof) between delay-period spiking or oscillatory activity within a single brain area and behavior is shown in Table 1; it includes both single-neuron firing rate studies, and population-level measures based on local field potentials (LFPs) in non-human primates or intracranial recordings in humans (ECoG). LFP activity, which reflects a combination of local activity and sub-threshold network input (Buzsáki et al., 2012; Friston et al., 2015), provides a window onto local oscillatory activity and synchronization between areas (for a review of EEG findings, see Fell and Axmacher, 2011). Proportionally, very few studies measuring persistent activity in single cells report a correlation with behavioral performance, and several fail to find such a performance correlation (Nakamura and Kubota, 1995; Siegel et al., 2009; Liebe et al., 2012; Rigotti et al., 2013; Sarma et al., 2015; Antzoulatos and Miller, 2016; Parthasarathy et al., 2017; Bahmani et al., 2018; Lundqvist et al., 2018; Rezayat et al., 2021). However, we should note that many studies measuring persistent spiking activity in non-human primates are not optimized for finding such behavioral correlations, since animals are extensively trained, often leaving few error trials for analysis (Pessoa et al., 2002). Meanwhile the publication bias against reporting negative results (i.e., a lack of behavioral correlation) will introduce bias in the opposite direction (Leavitt et al., 2017). The role of persistent spiking activity in the maintenance of WM is an active subject of debate in the field (Dedoncker et al., 2016; Christophel et al., 2017; Leavitt et al., 2017; Constantinidis et al., 2018; Miller et al., 2018); some activity-silent theories of WM depend on changes in synaptic weights rather than ongoing spiking activity (Mongillo et al., 2008; Stokes, 2015), which would certainly explain the lack of a strong behavioral correlation for delay period spiking activity. Table 1 summarizes cases where behavioral correlations of spiking or LFP activity are reported.

Population-level signatures (such as LFP) are more likely than single neuron activity to predict WM performance (see Table 1). The LFP power spectrum provides a representation of oscillatory activity in different frequency bands, which may also relate to the relative timing of activity within an area or to fluctuations in synaptic input (Buzsáki et al., 2012). WM modulation of LFP power has been reported in the prefrontal (Lundqvist et al., 2016, 2018), parietal (Pesaran et al., 2008), and sensory areas (Siegel et al., 2009; Barr et al., 2010; Helfrich and Knight, 2016; Lundqvist et al., 2016, 2018) of monkeys and in the prefrontal cortex (Howard et al., 2003; Noy et al., 2015; Johnson et al., 2018a), hippocampus (van Vugt et al., 2010; Ni et al., 2017), medial temporal lobe (Howard et al., 2003; Courtney, 2008; Ni et al., 2017; Johnson et al., 2018a), sensory areas (Noy et al., 2015), and parietal cortex (Noy et al., 2015) of humans. Within PFC, γ band activity increased during encoding and retrieval of information but decreased during the delay period, while β band power showed the opposite pattern (Lundqvist et al., 2016, 2018); deviation from this pattern of activity predicted errors (Lundqvist et al., 2018). Within parietal cortices, both spiking activity and the γ band LFP power during the delay predicted the animal’s choice (Pesaran et al., 2002). In sensory areas which lack persistent spiking activity, αβ band LFP power increased during the delay period of a WM task (Mendoza-Halliday et al., 2014; Bahmani et al., 2018). This change in power was correlated with performance (Bahmani et al., 2018) and reflected the content of WM (Mendoza-Halliday et al., 2014).

In addition to modulating power in different LFP frequency bands, WM also alters oscillatory synchronization, and spike timing relative to these oscillations within an area [often measured via Phase-Phase Locking (PPL) or Spike Phase Locking (SPL), respectively]. Oscillatory synchrony within sensory areas was measured by phase locking between different sites within MT (Tallon-Baudry et al., 2001; Bahmani et al., 2018), V4 (Tallon-Baudry et al., 2004), or IT (Tallon-Baudry et al., 2001, 2004), all of which showed increases in αβ band phase locking during WM. Phase-amplitude coupling (PAC) measures interactions between different frequency bands (more specifically, the phase of one frequency and the amplitude of another, typically higher frequency); WM induces changes in PAC in the hippocampus (Axmacher et al., 2010), and across different layers of PFC (Bastos et al., 2018) and MT (Maris et al., 2011). In PFC, the αβ phase in the deep layers modulated γ band activity in the superficial layers (Bastos et al., 2018). Within PFC and sensory areas, the maintenance of WM is accompanied by modulation of SPL in the β and θ bands (Siegel et al., 2009). Interestingly, the specific phase values at which spikes were locked predicted the content of WM and behavior (Siegel et al., 2009). Similarly, in MT, average spiking activity didn’t reflect the content of WM, but the SPL in the αβ band did (Bahmani et al., 2018). Moreover, greater SPL during WM corresponded with enhanced processing of sensory input (Bahmani et al., 2018). Similarly, WM information in spiking activity varied with the θ cycle in area V4 (Lee et al., 2005). As described, SPL analysis provides a widow onto the temporal coding of spiking activity, which sometimes reveals information not detectable in rate coding over longer time windows. These phase locking measures within an area are also more likely than the firing rate of individual neurons to correlate with performance.

## Synchronization Between Areas During Working Memory Predicts Performance

**FIGURE 1:**
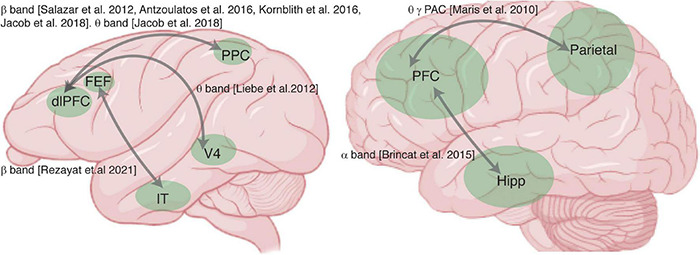
Summary of studies examining synchrony between areas during working memory (WM). Brain schematics of the monkey **(*left*)** and human brain **(*right*)**, and areas recorded (green) in studies reporting measurements of synchrony between areas. Gray arrows indicate areas recorded in the same study, labeled with the frequency band in which WM-induced changes in synchrony between the areas were reported.

Many areas have delay activity during WM (Christophel et al., 2017; Leavitt et al., 2017; Sreenivasan and D’Esposito, 2019), raising the question of whether interactions between these areas contribute to memory maintenance. Here we review evidence that changes in synchrony between areas occur during WM: there is evidence for PFC interacting with sensory areas, the parietal cortex, and the hippocampus during WM (summarized in Figure 1).

Let’s begin by briefly outlining three conceptual models which describe the role of PFC’s interactions with sensory cortex, parietal cortex, and hippocampal areas during WM: *sensory recruitment*, *distributed network*, and *activation of long-term memory* models. In all of these models the PFC is believed to be crucial, based on extensive literature on its role in executive function (Jacobsen and Nissen, 1937; Piaget, 1964; Niki, 1974; Kojima and Goldman-Rakic, 1982; Funahashi et al., 1989; Miller et al., 1996). The sensory recruitment model seeks to describe the interaction between PFC and sensory areas during WM (Pasternak and Greenlee, 2005), suggesting that sensory areas maintain detailed sensory memories under control of the PFC. Distributed network models of WM posit that interactions between association areas (such as parietal and prefrontal cortex) are necessary for memory maintenance (Leavitt et al., 2017). In the activation of long-term memory theory, PFC-hippocampal interactions maintain WM via activation of long-term memory representations (Eriksson et al., 2015; Loaiza and Halse, 2019; Rhodes and Cowan, 2019); it has also been suggested that information must pass through WM before entering long-term memory (Eriksson et al., 2015; Loaiza and Halse, 2019; Rhodes and Cowan, 2019). Synchrony between the PFC and areas associated with long-term memory (such as the hippocampus) could reflect either of these processes. In all of these scenarios (sensory recruitment, distributed network, or activation of long-term memory representations), synchronized activity across areas plays a key role in WM tasks (Leszczyński et al., 2015). In the following paragraphs we discuss some evidence for each of these interactions.

**FIGURE 2:**
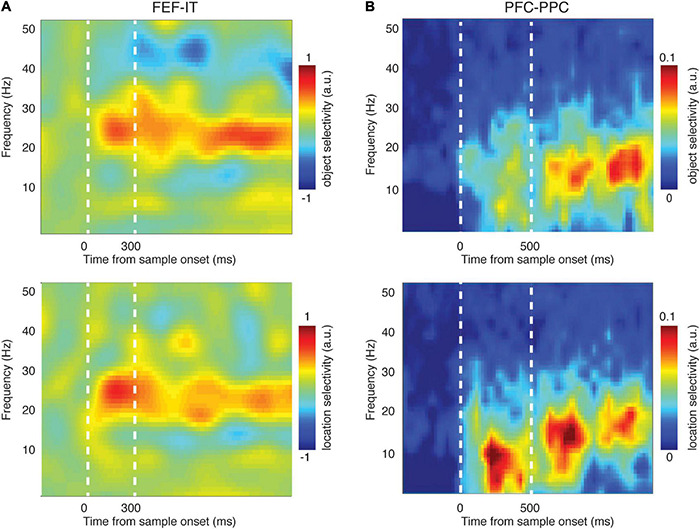
Synchronized activity between brain areas reflected the content of WM. **(A)** Phase-phase locking (PPL) between PFC (FEF) and temporal cortex (IT) encoded the identity (top) and location (bottom) of the sample object during a delayed-match-to-sample task [adapted from Rezayat et al. (2021)]. Heatmap shows the difference in PPL between conditions (different object identities or locations) across time and frequency. **(B)** LFP-LFP coherence between PFC and parietal cortex encoded the identity (top) and location (bottom) of the sample object during the delayed-match-to-sample task [adapted from Salazar et al. (2012)]. Heatmap shows the difference in coherence between conditions (different object identities or locations) across time and frequency.

First, several studies report synchrony between PFC and sensory areas during WM. Phase synchrony between PFC and temporal cortex increased during memory maintenance (Liebe et al., 2012; Rezayat et al., 2021). Figure 2 shows that synchrony between brain areas reflects the content of WM. Phase locking between PFC and IT, specifically in the β band, reflected both the identity and the location of a remembered object (Figure 2A). These inter-areal synchrony measures were also related to WM performance (Table 2). Synchrony between PFC and V4, specifically in the θ band, predicted memory performance (Liebe et al., 2012), as did β band synchrony between PFC and IT cortex (Rezayat et al., 2021). This inter-areal phase locking and spike-phase synchrony was correlated with performance even when within-area signatures showed little or no relationship to memory performance (Rezayat et al., 2021).

**TABLE 2:** Neural signatures of WM between areas and their relationship to behavior.

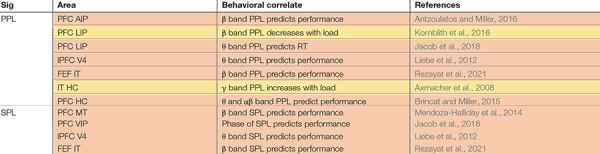

*Studies are grouped according to the neural signature being studied (Sig), then by the area being recorded from Area. The relationship between a particular neural signature and the animal’s behavior on a WM task (performance, RT, or WM load) is noted (Behavioral correlate), for the specified frequency band (unspecified bands showed no such correlation). Each row corresponds to one publication (References). Studies which report data for more than one area or signature may be listed multiple times. Color coding and abbreviations as in Table 1.*

There are many studies suggesting that the content of WM is maintained via the interaction of association areas across a distributed network (Christophel et al., 2017; Leavitt et al., 2017); indeed, the frontoparietal network is thought to play a key role in a variety of cognitive functions (Sarnthein et al., 1998; Zaksas et al., 2001; Babiloni et al., 2004; Olesen et al., 2004; Freedman and Assad, 2006; Hamidi et al., 2008; Salazar et al., 2012; Zhou et al., 2012; Dotson et al., 2014, 2018; Antzoulatos and Miller, 2016; Rose et al., 2016; Mackey and Curtis, 2017; Violante et al., 2017; Jacob et al., 2018; Wang S. et al., 2019), including WM. Phase synchrony between prefrontal and parietal cortex, specifically in the β band, reflected the content of WM (Salazar et al., 2012; Dotson et al., 2014; Antzoulatos and Miller, 2016; Figure 2B). Frontoparietal β band synchrony also predicted WM performance (Antzoulatos and Miller, 2016). In human intracranial recordings, frontoparietal delta and θ band oscillations modulated WM representations (Johnson et al., 2018b). Much of the evidence for the involvement of frontoparietal synchrony in WM comes from human EEG and MEG research, including evidence of phase synchronization (Babiloni et al., 2004; Deiber et al., 2007; Rutman et al., 2010; Zanto et al., 2011) and phase-amplitude coupling (Reinhart and Nguyen, 2019). Cross frequency coupling measured by PAC in human ECoG is observed between many brain areas (Bruns and Eckhorn, 2004; Maris et al., 2011) and these interactions are related to frontoparietal connectivity (Figueroa-Vargas et al., 2020). All of this evidence points toward a role for frontoparietal synchrony during WM.

The role of long-term memory in WM (Jensen and Lisman, 2005) remains controversial. The observation of prefrontal-hippocampal synchrony during WM maintenance (Brincat and Miller, 2015) seems consistent with the suggestion that the hippocampus supports WM by activating long-term memory representations (Eriksson et al., 2015; Loaiza and Halse, 2019; Rhodes and Cowan, 2019). In support of recruitment of the hippocampus during WM, there is evidence for an increase in the γ band power within PFC and the hippocampus during WM (Brzezicka et al., 2019), and imaging studies have shown an interaction between PFC and the hippocampus during WM tasks (Gluth et al., 2015; Calabro et al., 2020). Interestingly, similar interactions between PFC and the hippocampus are observed during the transformation of information from short-term memory to long-term memory during sleep (Helfrich et al., 2019) [Another version of the long term memory activation hypothesis suggests that prefrontal-parietal synchrony reflects an attentional pointer to information stored in long term memory (Ruchkin et al., 2003)]. Studies on the effect of hippocampal lesions on WM performance somewhat complicate the picture. Both human clinical studies (Spiers et al., 2001) and induced hippocampal lesions in monkeys (Zola et al., 2000) indicate that the hippocampus is not necessary for simple WM performance with short delays; however, there is evidence that the hippocampus contributes to short-term *spatial* memory (Jarrard, 1993; Kessels et al., 2001), and to more complex WM tasks requiring higher-order binding or associations (Yonelinas, 2013). This suggests that the prefrontal-hippocampal interactions observed during WM serve a purpose other than simple WM maintenance, perhaps contributing to maintaining more complex associations or bindings within WM, in addition to potentially reflecting the transfer of information to long term memory.

## Dysfunctions in Oscillations and Synchrony During Working Memory Occur in Brain Disorders

If oscillations and synchrony are important for normal brain function, one might expect them to be disrupted in various mental disorders, and this is indeed the case (Uhlhaas and Singer, 2006). These changes in synchrony are important not only for understanding the mechanism of the underlying pathology, but also as a potential non-invasive biological diagnostic (Gandal et al., 2012), which may be detectable early in the disease process, and for developing treatments (Strüber and Herrmann, 2020). As a core cognitive function, the impairment of WM appears in many different disorders including schizophrenia (Park and Holzman, 1992; Goldman-Rakic, 1994; Roitman et al., 2000; Peled et al., 2001; Kim et al., 2003; Micheloyannis et al., 2006; Basar-Eroglu et al., 2007; Haenschel et al., 2007, 2009; Badcock et al., 2008; Pachou et al., 2008; Barr et al., 2010, 2017; Griesmayr et al., 2014; Lett et al., 2014; Wu et al., 2014; Pinal et al., 2015; Senkowski and Gallinat, 2015; Van Snellenberg et al., 2016; Kang et al., 2018; Ma et al., 2018; Appaji et al., 2020; Murphy N. et al., 2020), bipolar disorder (Wu et al., 2014; Appaji et al., 2020), autism spectrum disorder (Bangel et al., 2014; Rabiee et al., 2018), Parkinson’s disease (Siegert et al., 2008; Cools and D’Esposito, 2011; Zhao et al., 2018; Harrington et al., 2020), psychosis (Gold et al., 2019), Attention-deficit/hyperactivity disorder (ADHD; Martinussen et al., 2005; Wolf et al., 2009; Matt Alderson et al., 2013; Bédard et al., 2014; Wu et al., 2017; Zammit et al., 2018; Jang et al., 2020), and depression (Shan et al., 2018). The role of synchronized oscillations in different brain disorders has been thoroughly examined elsewhere (Uhlhaas and Singer, 2006), and there is much recent interest in identifying non-invasive and quantitative signatures for different disorders (Başar, 2013; Nimmrich et al., 2015; Asllani et al., 2018). Here we provide a brief overview of some key lines of research related to changes of inter-areal synchrony in disorders affecting WM. Desynchronization across brain areas has been reported for schizophrenia (Peled et al., 2001; Kim et al., 2003; Micheloyannis et al., 2006; Haenschel et al., 2007; Pachou et al., 2008; Griesmayr et al., 2014; Lett et al., 2014), autism spectrum disorder (Bangel et al., 2014), Parkinson’s disease (Siegert et al., 2008), ADHD (Zammit et al., 2018; Jang et al., 2020) and psychosis (Hudgens-Haney et al., 2017). Abnormal cortical synchrony in PPL (measured with EEG) has been reported in schizophrenia during WM within dorsolateral prefrontal (Kang et al., 2018), posterior parietal (Kang et al., 2018), and visual cortices (Kang et al., 2018; for review see Brennan et al., 2013). Imaging-based connectivity measures showed lower connectivity between cortical areas in schizophrenia (Kim et al., 2003). Autism groups have less β band synchronization across multiple brain areas, as measured by MEG (Bangel et al., 2014). In the autism spectrum disorders and Williams syndrome there is reduced β band coherence and stronger γ band oscillations during perceptual tasks (Castelhano et al., 2015). ADHD is associated with a decrease in functional connectivity across prefrontal and parietal cortex (Wolf et al., 2009; Bédard et al., 2014). There is a significant different in θ band phase synchrony across the frontoparietal network in the ADHD group, measured by EEG (Jang et al., 2020). In an animal model of schizophrenia, globally administering an *N*-methyl-D-aspartate receptor antagonist, WM was impaired; this WM disruption was accompanied by enhanced α and low-γ band power, and dampening of the β band oscillations in the lPFC, both during the delay period and between trials (Ma et al., 2018). Additionally, WM deficits are accompanied by poor interregional synchrony in rodent models of schizophrenia (Sigurdsson et al., 2010). However, optogenetically inducing delta oscillations in the thalamic projection to the hippocampus impairs WM performance in rodents- so not all oscillatory manipulations are beneficial (Duan et al., 2015; Rahman et al., 2021). In summary, a variety of brain disorders characterized by WM impairments also show evidence of changes in synchronization between brain areas, supporting the hypothesis that such synchronization is important for WM performance.

## Manipulation of Inter-Areal Synchrony Alters Working Memory Performance

Whether synchrony and oscillations have a role in information processing in the brain, or are primarily epiphenomenal, has long been a subject of debate. The best way to test the functional role of synchrony is through causal experiments that selectively alter synchronous activity across brain areas. This is most directly accomplished by simultaneously manipulating activity across multiple areas (although manipulations of one area sometimes have indirect effects on synchrony). In comparing the frequency of changes in WM performance for studies manipulating activity in just one vs. multiple areas (Table 3), we observe that those manipulating multiple areas more reliably impacted WM performance, consistent with an important functional role for inter-areal synchrony in WM performance. Effects of the manipulations on performance are summarized in Table 3, and effects on brain activity, oscillations, or synchrony in Table 4.

**TABLE 3:** Causal manipulations of oscillations or synchrony and their effect on WM.

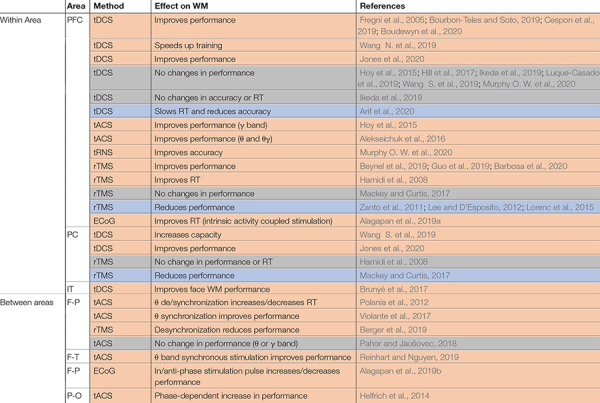

*Studies are grouped according to whether they include one or multiple areas, then by the area(s) being manipulated (Area). The method of manipulation is specified (Method), along with the effect on WM, and relevant citations (References). Coloring indicates whether the manipulation impacted behavior on a WM task (performance, RT, or training time); rows in orange showed improved performance or RT, gray showed no effect, and blue indicates a detrimental effect on performance or RT. Performance indicates percent correct trials. PC, parietal cortex; F-T, frontotemporal; F-P, frontoparietal; P-O, parieto-occipital; tRNS, transcranial random noise stimulation.*

**TABLE 4:** Causal manipulations of oscillations or synchrony and effect on neural measurements.

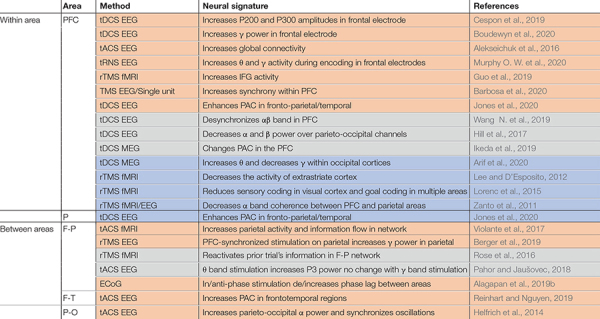

*Studies are grouped according to whether they include one or multiple areas, then by the area(s) being manipulated (Area). The method of manipulation and measuring brain activity is specified (Method), along with the effect on neural activity (Neural Signature), and relevant citations (References). Color coding reflects behavioral effect, as in Table 3: for rows in orange stimulation improved performance or RT, gray showed no effect, blue indicates a detrimental effect on performance or RT. IFG, inferior frontal gyrus; P-T, parieto-temporal.*

Manipulations which can alter oscillatory activity in a single area are sometimes sufficient to impact WM performance (Table 3), as well as modulating activity elsewhere in the brain (Table 4). Manipulations are carried out using a variety of techniques, as indicated in the third column of each table. Electrical stimulation with subdural electrodes over the superior frontal gyrus, in the same frequency range as endogenous activity during WM (θ -α), reduced subjects’ reaction times (Alagapan et al., 2019a). Transcranial direct current stimulation (tDCS) applies a low DC current to pairs of electrodes placed on the head. There are mixed results for the effect of tDCS over prefrontal cortex. Some studies reported that tDCS boosted WM performance when applied over the PFC (Fregni et al., 2005; Jones et al., 2015; Bourbon-Teles and Soto, 2019; Cespon et al., 2019; Wang S. et al., 2019; Boudewyn et al., 2020), while others showed no effect or reduced performance (Hoy et al., 2015; Hill et al., 2017; Ikeda et al., 2019; Luque-Casado et al., 2019; Wang S. et al., 2019; Murphy O. W. et al., 2020). Within sensory areas, tDCS of right fusiform regions involved in face representation and memory specifically enhanced WM performance for faces but not scenes (Brunyé et al., 2017). Transcranial magnetic stimulation (TMS), which applies a high intensity magnetic pulse on the area under a coil, also has mixed impacts on WM performance. TMS over PFC can improve WM performance (Hamidi et al., 2008; Beynel et al., 2019; Guo et al., 2019; Barbosa et al., 2020), but not always (Zanto et al., 2011; Lee and D’Esposito, 2012; Lorenc et al., 2015). Another technique, transcranial alternative current stimulation (tACS), uses an AC current (at some specific frequency) rather than a DC current, allowing intentional targeting of specific oscillatory frequencies. With this method the frequency of stimulation can be chosen to match the frequency of intrinsic oscillations recorded at the site, which can increase the effect of the manipulation on performance (Hoy et al., 2015; Alekseichuk et al., 2016; Murphy O. W. et al., 2020).

In spite of the popularity of tDCS, tACS, and TMS, the exact effect of these types of stimulation on brain activity remains a topic of investigation (Liu et al., 2018). tACS can modulate oscillatory activity (Liu et al., 2018). tDCS increased neural excitability and spontaneous activity in the area under the anodal electrode (Rahman et al., 2013; Krause et al., 2017; Liu et al., 2018; Kunori and Takashima, 2019). TMS increased spiking activity and selectivity in the affected region (Pasley et al., 2009; Mueller et al., 2014; Kim et al., 2015; Romero et al., 2019). Note that although tDCS and TMS do not directly induce a particular oscillatory frequency based on the stimulation parameters, they can nevertheless sometimes produce frequency-specific changes in power within the stimulated area (Boudewyn et al., 2020) or elsewhere in the brain (Ni et al., 2016; Hill et al., 2017), and alter oscillatory synchrony between areas (Zanto et al., 2011; Jones et al., 2020). TMS of one area can also modulate processing elsewhere in the brain. TMS of PFC modulated the fidelity of information in visual cortex (as measured by patterns of fMRI activity) during a WM task (Lorenc et al., 2015). Disrupting PFC with TMS during memory encoding diminished top-down modulation of activity in posterior cortex during encoding, which predicted the subsequent decrement in WM accuracy (Zanto et al., 2011; Lee and D’Esposito, 2012). TMS over parietal cortex can reactivate the latent content of WM, as measured by performance and patterns of fMRI activity (Rose et al., 2016). These studies show that manipulation in one part of the network can be sufficient modulate neural activity and impact task performance.

Manipulation of neural activity in multiple areas has also been used to more directly control the synchrony between areas, with more reliable effects on behavioral performance. Synchronized θ band tACS of frontoparietal networks enhanced performance on a demanding verbal WM task (Violante et al., 2017). This stimulation increased parietal activity (measured via fMRI), which correlated with behavioral performance. Synchronous stimulation of both sites was critical for this effect; stimulation of prefrontal cortex alone did not improve performance in the same study (Violante et al., 2017), nor was performance improved by prefrontal or parietal θ or γ band stimulation in another study evaluating visual WM (Pahor and Jaušovec, 2018). Intracranial stimulation of two areas in the frontoparietal network showed a similar effect: in-phase stimulation decreased phase lag between areas and enhanced WM performance, but antiphase stimulation increased the phase lag between areas and had no effect on performance (Alagapan et al., 2019b). tDCS of the frontoparietal network enhanced WM performance and increased coupling between the θ band phase in PFC and the γ band amplitude in parietal cortex (Jones et al., 2020). Synchronized θ band stimulation of frontal and temporal areas increased subsequent synchrony between areas (measured with EEG), and enhanced WM performance in the elderly (Reinhart and Nguyen, 2019). Neither prefrontal nor temporal stimulation alone, nor asynchronous stimulation of both areas, improved performance in the same study. In another recent study, the time of stimulation was controlled relative to the phase of endogenous activity in another area (Berger et al., 2019). The timing of TMS stimulation of parietal cortex (relative to the phase of the θ oscillation in frontal cortex) determined whether it enhanced or suppressed parietal γ activity. Stimulation at the trough of the frontal θ oscillation enhanced the γ band activity and performance; stimulation at the θ peak had the opposite effect (Berger et al., 2019). With appropriate tACS intensity and frequency, synchronous activity across areas can be manipulated (Rahman et al., 2013; Krause et al., 2017; Liu et al., 2018). Lower frequency stimulation with higher field intensity can impose synchrony in the network (Liu et al., 2018), and some tACS has been shown to modulate the timing of spiking activity rather than firing rate in primates (Krause et al., 2019). As one example of the power of this approach, such modulation of synchrony can control epileptic activity (Bikson et al., 2001; Sunderam et al., 2006; Berényi et al., 2012; Desai et al., 2016; Asllani et al., 2018).

Although neurophysiological findings have recently shown modulations of β band synchrony during WM (Siegel et al., 2009; Salazar et al., 2012; Dotson et al., 2014; Lundqvist et al., 2016; Bastos et al., 2018; Zanos et al., 2018), and modulations in this band are often predictive of WM performance (Tallon-Baudry et al., 2004; Siegel et al., 2009; Mendoza-Halliday et al., 2014; Brincat and Miller, 2015; Antzoulatos and Miller, 2016; Bahmani et al., 2018; Lundqvist et al., 2018; Rezayat et al., 2021), this frequency range has yet to be used in stimulation studies examining WM. However, note that β band stimulation is common during motor tasks, reviewed in Wischnewski et al. (2019). Microstimulation or optogenetic manipulations in animals hold the potential to modulate synchrony in a more spatially precise manner, testing the role of synchrony in maintaining specific representations. Indeed, rodent studies have reported selective modulation of synchrony by optogenetic methods (Kidder et al., 2021; Quirk et al., 2021). Once we better understand the role of synchrony, the ability to selectively manipulate it could provide treatments for neural disorders (Sreeraj et al., 2019).

## Role of Synchrony in Working Memory

A variety of functions have been proposed for inter-areal synchrony, which we briefly survey here before introducing our own interpretation specific to its role in WM. Synchrony between areas is widely hypothesized to alter the efficacy of communication or information transfer between them (Fries, 2015); this has been most extensively studied for the γ band (for review see Hahn et al., 2019). However, many neural signatures of WM involve coupling in the β or θ band (detailed above), and the role of these frequencies in modulating the efficacy of communication remains comparatively less explored. One possibility is that lower frequency synchronization can provide a temporal framework for higher frequency synchrony (Sauseng et al., 2009; Siebenhühner et al., 2016); PAC measures may reflect the nesting of γ band oscillations within lower frequency oscillations, as observed in WM studies (Maris et al., 2011; van der Meij et al., 2012; Jiang et al., 2015). For β band synchrony two main roles have been suggested (Miller et al., 2018). One is that β band synchrony links the phase of deep layers of PFC to the γ band oscillation in the superficial layers (Bastos et al., 2018). In this scenario the β band is a source of internal inhibitory control, and the power in the β and γ bands are anticorrelated (Lundqvist et al., 2016, 2018; Bastos et al., 2018). Another potential role of β oscillations, proposed based on modeling results, is that they underlie the formation of dispersed neuronal ensembles (Kopell et al., 2011), and ensemble activity could represent the content of WM. A third possible role of β oscillations is to help drive changes in synaptic weights: the timing of neural activity relative to β oscillations has been shown to affect synaptic plasticity (Zanos et al., 2018), and plasticity is the basis of activity-silent WM models (Mongillo et al., 2008; Stokes, 2015).

**FIGURE 3:**
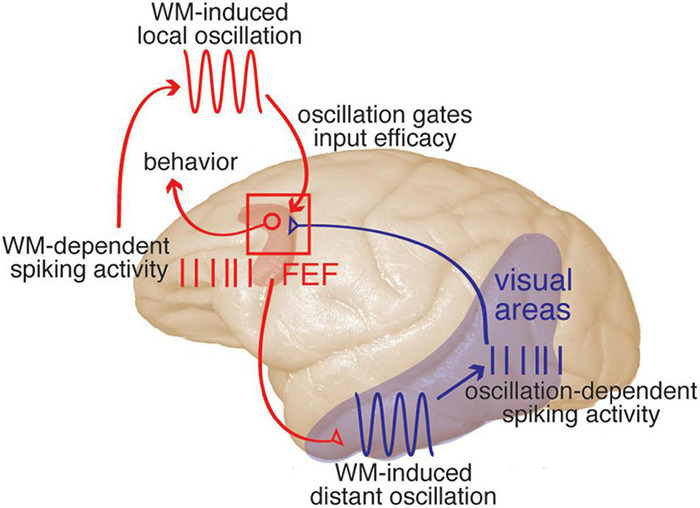
A framework describing prefrontal-visual interactions and the functional role of coordinated oscillations during WM. Description moves counter-clockwise from upper left. *WM-dependent spiking activity*: spiking activity within PFC (red) represents the content of WM. This activity is sent from PFC to visual areas via direct projections (red projection), recruiting sensory areas. *WM-induced distant oscillation*: within visual areas (blue), the top-down WM input drives an αβ-frequency oscillation. *Oscillation-dependent spiking activity*: the combination of this αβ oscillation and a neuron’s sensitivity to sensory input will determine its spike timing relative to the local αβ oscillation. *S*pikes are sent from visual areas to PFC (blue projection). *WM-induced local oscillation*: WM activity within PFC also drives an αβ oscillation within PFC, which will be phase-locked with that in visual areas. *Oscillation gates input efficacy*: the phase of the αβ oscillation within PFC will gate the efficacy of visual input, providing a mechanism to preserve the information contained in spike timing relative to the oscillation. Visual inputs to PFC target visuomotor neurons, and evoked activity in PFC will in turn guide behaviors (for example, eye movements), with the net result that incoming stimuli matching the content of WM are more likely to influence behavior. This model is based on results that have been reported in one or more visual areas including V4, MT, and IT, for spatial or object WM (see text for references).

Here we introduce an overall framework for the interactions between PFC and visual cortex during WM (Figure 3), which explains a constellation of experimental results, including the close tie between signatures of inter-areal coordination and WM performance. A full description of the theoretical framework and all the relevant literature will require an entire separate review article; here we provide an outline of the key concepts and most relevant literature.

Overall, the emerging picture is that PFC spiking activity representing the content of WM is sent to visual areas during WM maintenance (Merrikhi et al., 2017). This PFC “persistent activity” sent to visual areas recruits them by increasing the power of αβ oscillations within those areas. The PFC-induced oscillation in visual areas modulates the likelihood and timing of action potentials based on the sensitivity of the neuron. This allows visual neurons to reflect their sensitivity to visual stimuli in the timing of their spikes relative to the local oscillation: more sensitive neurons generate spikes earlier than less sensitive neurons in response to the WM-induced oscillation. This sensory information in the timing of spikes can be read out via a phase code when considering the timing of spikes relative to the phase of αβ oscillations (Bahmani et al., 2018). This information, encoded in the phase of spikes relative to the WM-induced αβ oscillation, can be decoded by electrophysiologists recording the spiking and LFP activity, but how can it be conveyed to downstream areas which receive only spiking activity? In order to access the information encoded in spike timing downstream areas must also have an oscillatory reference. Multiple groups have reported an increase in PPL between oscillations in prefrontal and visual areas during WM, as reviewed above (e.g., Liebe et al., 2012; Mendoza-Halliday et al., 2014; Daume et al., 2017; Rezayat et al., 2021). It is likely that the same source (i.e., persistent PFC activity) that drives the αβ oscillation in visual areas also generates a similar, coherent oscillation in areas receiving input from those visual areas. Thus, in this perspective, the inter-areal PPL observed during WM is a signature of sharing a similar oscillatory frame of reference. Another study from our group, tracing the fate of visual input to the FEF during WM, provides a clue to a potential mechanism for reading out the information contained in the timing of incoming spikes: the efficacy of visual inputs to PFC neurons increases at the location held in WM (Noudoost et al., 2021). Building on this observation, we propose that the coherent oscillation in the receiving area allows dynamic gating of arriving spikes, such that spikes arriving at a certain phase will more effectively drive the post-synaptic neurons; indeed, such changes in the sensitivity of an area to input based on the phase of local oscillations have previously been reported, albeit in the gamma band (Cardin et al., 2009; Knoblich et al., 2010; Yonelinas, 2013; Ni et al., 2016), and are consistent with the phase coding observed within PFC during WM (Siegel et al., 2009). Thus, the phase locking between visual and prefrontal areas during WM (e.g., Rezayat et al., 2021) is a signature of a shared oscillatory frame of reference, which both controls the relative timing of spike generation in visual areas and dynamically gates visual input efficacy in prefrontal areas—encoding and decoding information, respectively, in spike timing relative to the oscillation. These modulations of spike timing within visual areas, in combination with a coherent oscillation in prefrontal areas, mean that incoming sensory stimuli matching the content of WM will be more likely to drive prefrontal activity, and thus to guide behavior. WM relies on this recruitment of sensory areas by prefrontal areas, and thus, having a shared oscillatory frame of reference between areas is critical for WM performance, as reviewed in this manuscript.

A schematic illustrating the key components of the proposed framework for the prefrontal recruitment of sensory areas during WM is shown in Figure 3. It should be noted that thinking within the suggested framework will refine our definitions of some concepts:

– *Representation* is the sensitivity of a neuron to current input, including subthreshold modulations not visible in extracellular recordings (e.g., spiking activity). The WM-induced oscillation can facilitate the expression of that representation in the form of spiking activity for the purpose of inter-areal communication.
– *Sensory recruitment* is the process of facilitating sensory areas to express their representation in the form of spiking activity, which enables WM to take advantage of these areas’ greater visual selectivity. In order to recruit extrastriate visual areas, the FEF part of prefrontal cortex directly sends these areas persistent WM-related activity, which drives an αβ oscillation within them.
– *Feedback*: Although the projections from PFC to visual areas directly convey only spiking activity, notably including the content of WM, the purpose of this feedback is not merely to replicate that information in visual areas, but rather to drive a coherent oscillation between the two areas. This feedback-induced shared oscillatory frame of reference enables both phase-dependent encoding of visual information in visual areas, and decoding of that information using oscillation-dependent input efficacy in PFC.

As noted, we have limited ourselves to an overview of the framework and the associated interpretation of phase locking between areas; a full and detailed survey of the evidence for each component of the proposed model is beyond the scope of the current review. This framework provides an answer to several puzzles in the literature of sensory recruitment by WM (and more broadly the top-down control of sensory signals). Why would FEF, which does not have strong feature selectivity, show persistent activity during various forms of WM (Clark et al., 2012)? We propose that this FEF activity serves to drive a common oscillatory frame of reference in both V4 and FEF. Why do sensory areas with sufficient feature selectivity to satisfy WM requirements only show very weak modulations in their firing rate during memory maintenance? In this framework, the representational enhancement in these areas can only be traced in relation to WM-dependent oscillations. How would aligning spikes to a certain oscillation in visual areas benefit sensory processing, if that phase information is not sent along with spikes to the next area? We suggest that the area receiving visual input also has a copy of the phase reference, as evidenced by both the coherence of the oscillations, and the timing of visual spikes relative to the phase of PFC oscillations. Finally, of course, the framework offers an explanation for the main focus of this review- the close link between inter-areal coherence and WM performance.

## Concluding Remarks

We have reviewed evidence that oscillatory coupling between areas is crucial for WM. A mechanistic framework for understanding the necessity of such coupling is briefly described (Figure 3). Certain aspects of the proposed framework have yet to be directly tested. For example, what is the circuit mechanism driving coherent oscillations in visual and prefrontal areas? Does the role of oscillations in controlling spike timing in visual cortex rely on the same cellular mechanism that gates the efficacy of inputs in prefrontal cortex? Do aspects of the proposed framework apply to prefrontal-visual interactions outside of WM? The answers to these questions hold the potential to transform our understanding of prefrontal control, sensory representation, and the role of inter-areal communication in cognitive tasks.

## Author Contributions

ER, KC, and BN wrote the manuscript. M-RD and BN contributed to the development of ideas and discussion.

## Conflict of Interest

The authors declare that the research was conducted in the absence of any commercial or financial relationships that could be construed as a potential conflict of interest.

## Publisher’s Note

All claims expressed in this article are solely those of the authors and do not necessarily represent those of their affiliated organizations, or those of the publisher, the editors and the reviewers. Any product that may be evaluated in this article, or claim that may be made by its manufacturer, is not guaranteed or endorsed by the publisher.
